# Supplementary material for: The Diverging Routes of BORIS and CTCF: An Interactomic and Phylogenomic Analysis
Source: Life (Basel). 2018 Jan 30;8(1):4. doi: 10.3390/life8010004 (PMC5871936; doi:10.3390/life8010004)
Supplement: Supplementary file 1 [file life-08-00004-s001.pdf]

The diverging routes of BORIS and CTCF: An interactomic and phylogenomic analysis

Kamel Jabbari, Peter Heger, Ranu Sharma and Thomas Wiehe

Supplementary material

Table S1: Results of Blastn of CTCF from Fly catcher (*Ficedula albicollis*) on birds DNA sequences from NCBI.

	Max score	Total score	Query cover	E value	Ident	Accession
Ficedula albicollis	691	691	99%	0	99%	XM_005057389.1
Pseudopodoces humilis	644	644	100%	0	93%	XM_014254020.1
Parus major	637	637	100%	0	92%	XM_015647591.1
Corvus cornix	630	630	100%	0	92%	XM_010396812.3
Serinus canaria	629	629	99%	0	92%	XM_018917407.1
Manacus vitellinus	585	585	100%	0	84%	XM_018073983.1
Lepidothrix coronata	584	584	100%	0	84%	XM_017815221.1
Pseudopodoces humilis	537	650	98%	0	93%	XM_014254022.1
Gavia stellata	535	636	96%	0	78%	XM_009809153.1
Haliaeetus albicilla	519	622	97%	0	76%	XM_009917251.1
Fulmarus glacialis	496	496	97%	3.00E-172	72%	XM_009580776.1
Zonotrichia albicollis	479	479	83%	1.00E-168	85%	XM_014268469.1
Melopsittacus undulatus	466	564	95%	9.00E-159	71%	XM_013128648.1
Tyto alba	465	564	98%	9.00E-159	69%	XM_009973007.1
Calypte anna	462	555	98%	1.00E-157	67%	XM_008497988.1
Phaethon lepturus	456	561	84%	4.00E-157	76%	XM_010289200.1
Nestor notabilis	456	456	98%	9.00E-156	67%	XM_010010226.1
Taeniopygia guttata	447	520	72%	1.00E-155	90%	XM_004177089.1
Lonchura striata domestica	432	487	71%	8.00E-151	91%	XM_021553588.1
Columba livia	444	534	97%	2.00E-141	66%	XM_021283608.1
Lepidothrix coronata	450	450	75%	1.00E-140	86%	XR_001874876.1
Aptenodytes forsteri	412	516	71%	1.00E-140	82%	XM_019470692.1
Chlamydotis macqueenii	405	504	72%	1.00E-138	80%	XM_010129219.1
Opisthocomus hoazin	406	511	71%	3.00E-138	82%	XM_009933922.1
Numida meleagris	422	514	97%	8.00E-138	63%	XM_021416918.1
Apteryx australis mantelli	403	584	72%	7.00E-137	81%	XM_013951392.1
Tinamus guttatus	405	512	71%	3.00E-136	80%	XM_010227722.1

Table S2: Summary information on genes present in the syntenic region of mouse.

Protein	Function	Gene start	Expression
Spo11	SPO11 meiotic protein covalently bound to DSB	172,977,700	high in male tissues
Rae1	ribonucleic acid export 1	173,000,117	high in male tissues
Rbm38	RNA binding motif protein 38	173,020,498	widely expressed
Ctcfl	CCCTC-binding factor	173,093,609	high in male tissues
Pck1	phosphoenolpyruvate carboxykinase 1, cytosolic	173,153,048	very low
Zbp1	Z-DNA binding protein 1	173,206,612	very low
Pmepa1	prostate androgen induced 1	173,224,458	high in male/female tissues

CTCFL locus of *Alligator mississippiensis*

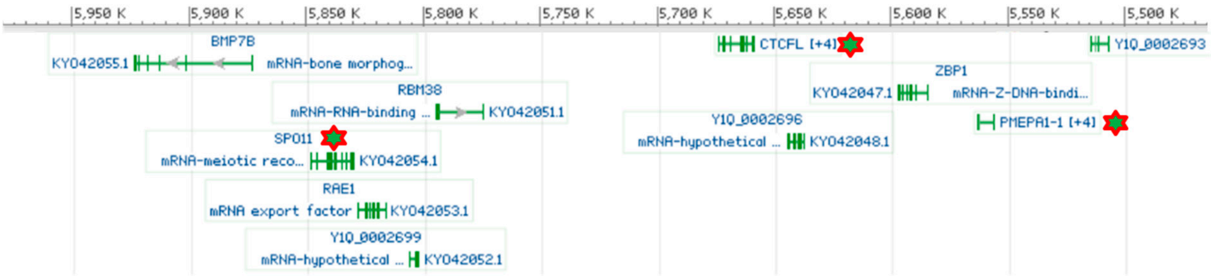

CTCFL locus of *Crocodylus porosus*

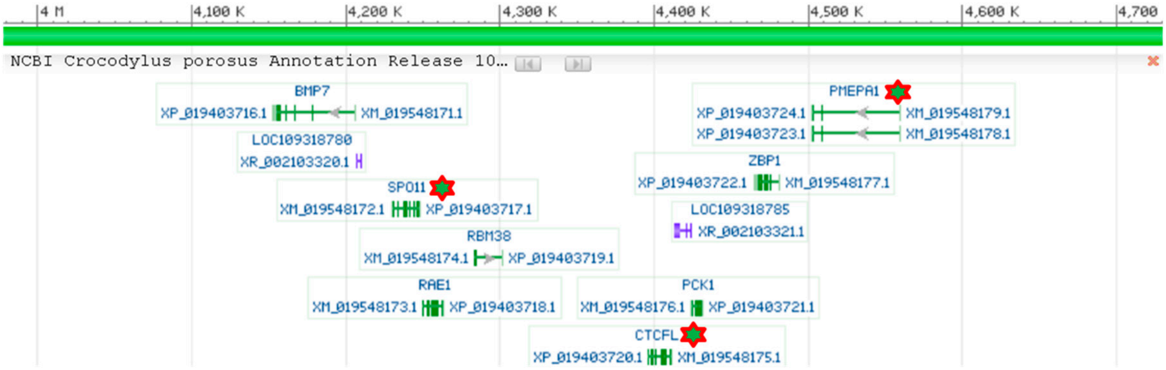

Figure S1: Locus Ensembl snapshot of conserved gene order around Spo11 in reptiles. Stars point to the conserved gene order alluded to in the main text (Spo11, CTCFL and PMEPA1).

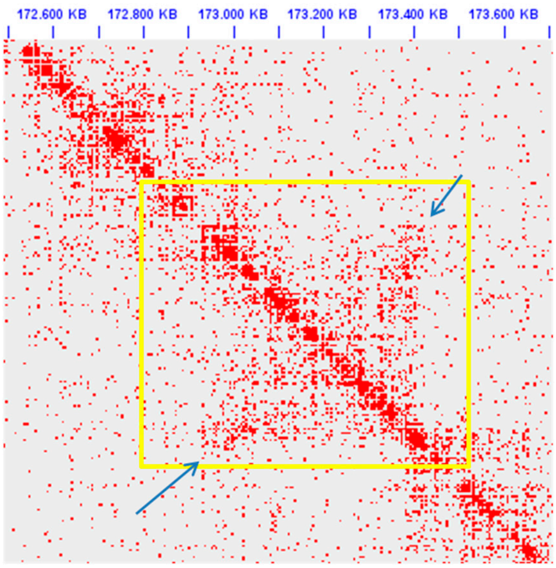

Figure S2: Mouse sperm cell Hi-C map from Battulin et al., showing the same loop as in Figure 6, Loop bases are indicated with arrows.

Figure S3

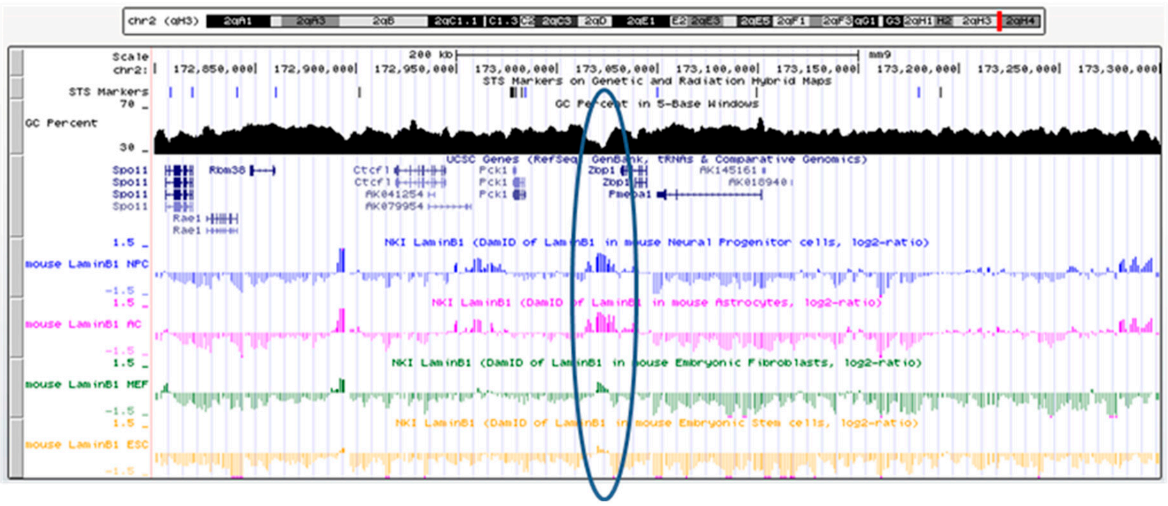

Figure S3: Snapshot of the UCSC mouse (mm9) genome browser showing lamin associated domains. Tracks show a high resolution map of the interaction sites of chromosome 2 (chr2:172,800,000-173,300,500) with Lamin B1 (a key Nuclear lamina component); the LADs state in sperm cell are not documented. ESC (embryonic stem cells); NPC (neural progenitor cells); AC (astrocytes); MEF (fibroblasts). The encircled region marks the possible repressive effect of LADs (lamina associated domains) on PCK1 and ZNP1.

Supplementary Materials
Section 1
Anolis lizard Gene: FAM131A ENSACAG00000002314

>Scaffold GL343245.1: 60,976-150,142 reverse strand.
PILMMVYSRVVIPTLCVIITLNSLFRATDQKVNVQCSEVLEAIENVILDVLKSLAKKKAP
VLTLANRSDWRNIEFKDSVGLQMIPHSSTKQIRSDCPATAPKFAMMLKILSMIYKMVQSN
SYATKRDIYYSDKLLFGSQRVVDNLINEISCMLQIPRRSLHILSTTRGFVAGNLSYTEED
GTKVNCTCGATAVTVPSNVQGIKNLYSHAKFILIVEKDATFQRLLDDEFCIKLAPCIMIT
GRGIPDLNTRLLVRKLWDTLQIPIFTLMDADPHGVEIMCIYKYGSVSMSFEAHQLTVPCI
KWLGLLPSDIKRLNIRKDVLIPFTKQDQNKLASLQKRPYIACQPVWKKELEIMAASKMKA
EIQVLTSLSSDYLSRVYLPNKLQFCGWI

Section 2

Uniprot annotation of human orthologs of nematode genes that were occasionally lost in parallel with CTCF.

RXRA_HUMAN: In the absence of ligand, the RXR-RAR heterodimers associate with a multiprotein complex containing transcription corepressors that induce histone acetylation, chromatin condensation and transcriptional suppression. On ligand binding, the corepressors dissociate from the receptors and associate with the coactivators leading to transcriptional activation. The RXRA/PPARA heterodimer is required for PPARA transcriptional activity on fatty acid oxidation genes such as ACOX1 and the P450 system genes. Plays a role in regulating enhancer activation (PubMed:28575647). Proposed core component of the chromatin remodeling INO80 complex which is involved in transcriptional regulation, DNA replication and probably DNA repair; proposed to target the INO80 complex to YY1-responsive elements.

SUZ12_HUMAN: Polycomb group (PcG) protein. Component of the PRC2/EED-EZH2 complex, which methylates 'Lys-9' (H3K9me) and 'Lys-27' (H3K27me) of histone H3, leading to transcriptional repression of the affected target gene. The PRC2/EED-EZH2 complex may also serve as a recruiting platform for DNA methyltransferases, thereby linking two epigenetic repression systems. Genes repressed by the PRC2/EED-EZH2 complex include HOXC8, HOXA9, MYT1 and CDKN2A
THB_HUMAN: Thyroid hormone receptor beta

TYY1_HUMAN: Multifunctional transcription factor that exhibits positive and negative control on a large number of cellular and viral genes by binding to sites overlapping the transcription start site. Binds to the consensus sequence 5'-CCGCCATNTT-3'; some genes have been shown to contain a longer binding motif allowing enhanced binding; the initial CG dinucleotide can be methylated greatly reducing the binding affinity. The effect on transcription regulation is depending upon the context in which it binds and diverse

mechanisms of action include direct activation or repression, indirect activation or repression via cofactor recruitment, or activation or repression by disruption of binding sites or conformational DNA changes. Its activity is regulated by transcription factors and cytoplasmic proteins that have been shown to abrogate or completely inhibit YY1-mediated activation or repression. For example, it acts as a repressor in absence of adenovirus E1A protein but as an activator in its presence. Acts synergistically with the SMAD1 and SMAD4 in bone morphogenetic protein (BMP)-mediated cardiac-specific gene expression (PubMed:15329343). Binds to SMAD binding elements (SBEs) (5'-GTCT/AGAC-3') within BMP response element (BMPRE) of cardiac activating regions. May play an important role in development and differentiation. Proposed to recruit the PRC2/EED-EZH2 complex to target genes that are transcriptional repressed. Involved in DNA repair. In vitro, binds to DNA recombination intermediate structures (Holliday junctions). Plays a role in regulating enhancer activation (PubMed:28575647). Proposed core component of the chromatin remodeling INO80 complex which is involved in transcriptional regulation, DNA replication and probably DNA repair; proposed to target the INO80 complex to YY1-responsive elements.

ZMYM2_HUMAN: Zinc finger MYM-type protein 2, May be a component of a BHC histone deacetylase complex that contains HDAC1, HDAC2, HMG20B/BRAF35, KDM1A, RCOR1/CoREST, PHF21A/BHC80, ZMYM2, ZNF217, ZMYM3, GSE1 and GTF2I.

ZMYM4_HUMAN: The 3'-UTR region of the mRNA encoding this protein contains a motif called CDIR (for cell death inhibiting RNA) that binds HNRPD/AUF1 and HSPB1/HSP27. It is able to inhibit interferon-gamma induced apoptosis.
